# Work Disability Trajectories Among Individuals with a Sick-Leave Spell Due to Depressive Episode ≥ 21 Days: A Prospective Cohort Study with 13-Month Follow Up

**DOI:** 10.1007/s10926-017-9751-9

**Published:** 2018-01-24

**Authors:** Kristin Farrants, Emilie Friberg, Sara Sjölund, Kristina Alexanderson

**Affiliations:** Division of Insurance Medicine, Department of Clinical Neuroscience, Karolinska Institutet, 171 77 Stockholm, Sweden

**Keywords:** Sick leave, Depression, Group-based trajectory modelling

## Abstract

*Background* Despite the increasing pattern of sick leave associated with depression in western countries, little is known about future work disability patterns among such sickness absentees. *Aim* To identify work disability (sick leave and disability pension) trajectories after the 21st day of a sick-leave spell due to depressive episode, and to investigate sociodemographic and morbidity characteristics of individuals in different trajectory groups. *Methods* This is a prospective cohort study using Swedish nationwide register data. We studied future work disability days (mean net days of sick leave and disability pension per month) among all individuals with a new sick-leave spell due to depressive episode (ICD-10 F32) ≥ 21 days during the first 6 months of 2010 (n = 10,327). Using group-based trajectory modeling, we identified work disability trajectories for the following 13 months. BIC value, group sizes, and average group probability were used to determine number of trajectories. Sociodemographic and morbidity characteristics were compared by χ^2^ tests. *Results* We identified six trajectories of work disability: “decrease to 0 after 4 months” (43% of the cohort); “decrease to 0 after 9 months” (22%); “constant high” (11%); “decrease, then high increase” (9%); “slow decrease” (9%); and “decrease, then low increase” (6%). Those in the groups “constant high” and “decrease then high increase” were older and had the highest proportion with sick leave the year before. *Conclusion* A majority of the cohort (65%) had no work disability by the end of follow up. Sociodemographic and morbidity characteristics differed between trajectory groups among people on sick leave due to a depressive episode.

## Background

Mental disorders have become leading causes of sick leave in OECD countries [1–3], and the prevalence of sick leave due to mental diagnoses is increasing [3–6]. People with a mental disorder have a higher risk of long-term sick leave and disability pension than those with a somatic diagnosis [4], even though most common mental disorders such as depression are treatable with current medical knowledge [7–9]. Long-term sick leave is a problem to employers and society in general as well as to absentees and their families [10–12]. For employers, it reduces productivity, for society it imposes sick-leave compensation costs, and for employees it can lead to financial and social difficulties [12–14] as well as to further morbidity and even mortality [15–17]. Individuals with longer sick leave are less likely to return to work, and more likely to leave the labour market via early retirement or disability pension, even when controlling for morbidity [12, 13, 18]. Sick leave and inactivity can have adverse consequences, both socially and for morbidity progression, especially for common mental disorders, including depression [7–9, 14].

From a clinical, employer, healthcare, societal, and patient perspective it would be helpful to have knowledge about how long a sick-leave spell that has just started might become, in order to plan for the future and introduce adequate interventions. Such predictions are difficult to make and physicians actually state this as one of their most problematic tasks in sickness certification consultations [19, 20]. In previous studies predicting duration of sick leave among people already on sick leave, the outcome is usually dichotomous, e.g., aiming at predicting if a sick-leave spell will be longer than a certain number of days or months [21–24]. However, more detailed knowledge is warranted about different absence patterns over time. The identification of groups of individuals with a similar trajectory of work disability can help identify factors that are associated with having a particular work disability trajectory at a more granular level than simply factors associated with work disability in general. This is important, as it can lead to having greater accuracy in early identification of people that are at risk of longer or recurrent work disability, and, thus, potentially to identification of individuals in need of early support or interventions. It is also of importance whether individuals who return to work, soon have a new sick-leave spell or even are granted disability pension. In Spain, those on short-term sick leave due to mental diagnoses were more likely to suffer a relapse of sickness absence than those with longer sick-leave spells [25]. More knowledge is, thus, needed on future patterns or trajectories of work disability (sickness absence and disability pension) among people on sickness absence.

Previous studies of people on sick leave regarding the risk of the sick-leave spell becoming long or sick-listed workers not returning to work have found that older age [26–32], female sex [27, 30], medium or high education [32], being on full-time sick leave [33], having other co-morbid conditions [34, 35], severity of depression [35], and previous sick leave [27, 29, 31, 36] were related to longer sick-leave duration and are risk factors of long-term sick leave, disability pension, or recurrent sick leave. A systematic review of factors associated with long-term sick leave among sick-listed employees (defined as at least 6 weeks, any cause) found that there was only evidence for old age and a previous history of morbidity and sick leave, and that the evidence for all other factors was weak or inconclusive [13]. A review of reviews for several diagnosis groups, not just mental, found that higher education or lower disease severity were positively associated with shorter work disability duration across diagnoses, while older age, female sex, higher pain or disability levels, depression, and previous sick leave were negatively associated with shorter work disability duration [37].

The duration of a sick-leave spell tends to vary with diagnosis [38], so what constitutes a long sick-leave spell for one diagnosis might be average, or even short, for another [32]. Also, for the same diagnosis, the severity of the condition may vary [11, 39]. Sick-leave spells due to mental diagnoses tend to be of longer duration than those due to somatic diagnoses [32, 40], and depressive episode has been linked to higher risk of disability pension [41, 42]. For depressive episode (ICD-10 code F32 [43]), the Swedish National Board of Health and Welfare’s guidelines for sick-leave certification [44] states that sickness absence in some cases might be needed up to a maximum of 180 days.

In Sweden, most of the sick-leave spells that last for > 14 days are due to mental diagnoses [45]. Depression commonly coexists with other mental and somatic conditions, and a Finish study found that those who have depression in addition to one or more other disorder have more frequent and longer sick-leave spells than those who have no other diagnosis than depression [34].

Laaksonen et al. [46] studied trajectories of preceding sickness benefit among a cohort of disability pensioners in Finland, and found four large groups with and two small groups without an increase of sick-leave days in the year preceding the disability pension. The two groups with no increase in sick-leave days differed from those who had, regarding sociodemographic factors and sick-leave diagnosis.

To the best of our knowledge, this is the first study to investigate trajectories of work disability among people sickness absent due to depressive episode. Rather than having a dichotomous outcome of return to work/work disabled at a particular time point, which may underestimate or overestimate work disability since return to work is a process that varies over time [47], studying trajectories of work disability enables us to gain knowledge on heterogeneity in both pace and extent (part- or full-time) of leaving work disability, by drawing on information from multiple time periods. This allows for more granular and detailed knowledge on variation in coming off of work disability by including several outcomes (trajectory groups), as well as treating work disability and return to work as a process.

In most countries, people with mental disorders have higher risk of not being employed. As Sweden has a public sickness benefit insurance, covering also people on unemployment benefits, a population-based study in Sweden would introduce less bias due to such exclusion mechanisms.

*The aim* of this study was to identify trajectories of work disability (sick leave and disability pension) after the 21st day of a sick-leave spell due to depressive episode (F32), and to investigate sociodemographic and morbidity characteristics of the individuals within the different trajectory groups.

## Methods

A prospective population-based cohort study was conducted, including all people aged 16–64 years and living in Sweden at 31 December 2009, who in the first 6 months of 2010 had a new sick-leave spell due to depressive episode (ICD-10 code F32 [43]) during the first 6 months of 2010, had not been on disability pension nor reached the maximum number of sick-leave days (365–914 days) in the year before, and who survived the first 180 days of the sick-leave spell (N = 10 327). For the 182 individuals who had more than one such sick-leave spell during those 6 months, the analyses were based on the first one. We call the sick-leave spell that led to inclusion in the cohort the index spell. The time limit of 21 days was chosen, as the first 14 days of a sick-leave spell are handled by the employer, and it can take an additional week before a Social Insurance officer gets involved.

We used data from the following five nationwide registers, linked at individual level by use of the Personal Identification Number (PIN, a unique ten-digit number assigned to all residents in Sweden): (1) Longitudinal Integration Database for Health Insurance and Labour Market Studies (LISA) held by Statistics Sweden (information on age, sex, country of birth, type of living area, family situation, and education); (2) MicroData for Analysis of the Social Insurance database (MiDAS) of the National Social Insurance Agency: sick leave and disability pension with benefits from the Social Insurance Agency [dates, extent (full-time or part-time), main diagnosis, and information on having reached maximum number of allowed sick-leave days (usually 914 days)]; (3) National Patient Register: morbidity [date and diagnoses of inpatient and specialized outpatient care (not primary healthcare)]; (4) the Prescribed Drug Register: purchased prescribed anti-depressive medication (ATC code: N06A); and (5) Cause of death register (dates). Registers 3, 4, and 5 are kept by the National Board of Health and Welfare.

All diagnoses were coded according to the International Classification of Diseases (ICD) version 10 [43]. The diagnosis of a depressive episode is to be used for the first onset of depression. If the patient has previously had a diagnosis of depression, the diagnosis of recurrent depression (ICD-10 code F33) is to be used, and thus, not included here. The sick-leave diagnoses in the MiDAS database are given at a three-digit level, e.g., F32, and is based on the information on the medical certificate, written by the treating physician. All physicians can issue sickness certificates, that is, not only the general practitioners [48]. The treating physician assesses the diagnosis, the level of function limitations it leads to, and possible level of work incapacity in relation to the patient’s work demands and thereafter writes the medical certificate [19, 49, 50]. The Social Insurance officer decides on whether the claimant fulfils the criteria for benefits and also investigates whether further actions are needed.

### Work Disability Insurance in Sweden

All people aged > 16 years, living in Sweden, and with an income from work or unemployment benefits are covered by the public sickness benefit insurance. Those who due to disease or injury have a reduced work capacity can receive sickness benefits [45]. After a first qualifying day, the employer pays sick pay for the first 14 days of a sick-leave spell, thereafter, sickness benefit is paid by the Social Insurance Agency. Self-employed have more qualifying days, and they as well as unemployed get all sickness benefits from the Social Insurance Agency. A certificate from a physician is required after 7 days of self-certification. All residents in Sweden aged 19–64 years, whose work capacity is permanently reduced due to disease or injury, can receive disability pension from the Social Insurance Agency. Since 2003, people aged 19–29 years can be granted temporary disability pension if their work capacity is reduced for at least 1 year and also regarding completion of upper-secondary education. Sickness benefits amount to 80% of lost income, disability pension to 65%, both up to a certain level. Sickness absence and disability pension can be for full-time or part-time (25, 50, or 75%) of ordinary working hours.

### Variables

The outcome variable was work disability, measured as mean number of net days of sick leave and disability pension per month (30-day period) in the 13 months, from the date of day 21 in the index sick-leave spell. For the calculation of net days, part-time work disability was combined, e.g., 2 days of 50% absence were combined to one net day, in order to handle that part-time absences are possible.

In the analyses, the following variables were included. How they are categorized is shown in Tables 1 and 3.

**Table 1:** Sociodemographic background characteristics for individuals with a new sick-leave spell due to depressive episode (F32) that lasted at least 21 days, proportion (%) of all and proportion (%) for which the sick-leave spell lasted for ≥ 180 days

	N	Proportion (%) of all	Proportion (%) with ≥ 180 days
All	10,327	100	28.7
Sex			
Women	7065	68.4	28.1
Men	3262	31.6	30.0
Age groups (years)			
16–24	619	6.0	22.0
25–34	2426	23.5	26.6
35–44	3115	30.2	30.5
45–54	2600	25.2	29.9
55–64	1567	15.2	28.9
Country of birth			
Sweden	8722	84.5	28.4
Other Nordic	306	3.0	22.9
Other EU25	258	2.5	26.7
Rest of world	1041	10.1	33.1
Education (years)			
Elementary (≤ 9)	1365	13.2	31.2
Secondary (10–12)	5170	50.1	27.7
Post-secondary (> 12)	3792	36.7	29.1
Type of living area			
Big cities (Stockholm, Gothenburg, Malmö)	4106	39.8	30.4
Medium-sized town (> 90,000 inhabitants)	3587	34.7	26.9
Small towns (< 90,000 inhabitants)	2634	25.5	28.4
Family situation			
Married/cohabiting without children	1070	10.4	25.8
Married/cohabiting with children	3855	37.3	28.6
Single/divorced/separated/widowed without children	3805	36.8	28.9
Single/divorced/separated/widowed with children	1555	15.1	30.5
≤20 years old living with parents	42	0.4	16.7
Marital status			
Single, divorced, widowed	6361	61.6	29.0
Married, registered partnership	3966	38.4	28.2
Employment status at start of index sick-leave spell			
Employed	8680	84.1	26.2
Unemployed	1092	10.6	46.9
Self-employed, student	338	3.3	36.4
Parental leave	217	2.1	24.9

#### Sociodemographic

Age, sex, country of birth, educational level, family situation, marital status, type of living area, employment status.

#### Sick-Leave Variables

Extent of the index sick-leave spell (full-time or part-time), any sick leave in the preceding 365 days, and diagnosis of previous sick-leave spell(s).

#### Prescribed Medication

Purchase of prescribed anti-depressive medication in the 12 months preceding the sick-leave spell, purchase of prescribed anti-depressive medication in the first 21 days of the index sick-leave spell.

#### Healthcare

Diagnosis and number of visits in outpatient specialized healthcare before and during the first 21 days of the index sick-leave spell, respectively. Diagnosis and number of days in inpatient care before and during the first 21 sick-leave days of the index spell, respectively. Healthcare due to normal childbirth (ICD-10 code O80) or for reasons other than disorders (ICD-10 codes Z00-99) were not included.

### Analysis

We present descriptive statistics of the study population and the proportion of spells that reached or exceeded 180 days, which is the maximum recommended sick-leave duration for F32 by the Swedish National Board of Health and Welfare.

We used a procedure known as Group-Based Trajectory Modelling (Proc traj in SAS) to identify possible trajectories of future work disability days (combining net sick-leave and disability pension days) during the following 13 months from the date of the 21st day of the sick-leave spell. We first determined the best-fitted model related to the number of groups using the Bayesian Information Criterion (BIC) [51], where values closer to 0 indicate better fit. The BIC value of a n group model was compared to a n−1 group model, and if the BIC value was smaller, the n group model was considered a better fit than the n−1 group model. In order to keep the number of groups within reach for interpretation, a requirement of a minimum of 5% of the study population for the smallest group was introduced [52]. We also used the method described by Coté et al. [53] to evaluate model fit, whereby the average probability for belonging to the group should be above 0.70 in all groups. The final model was the one that fulfilled all the criteria of decreasing BIC value, at least 5% of the study population in each group, and a ≥ 0.70 average probability of belonging to the group in all groups.

We used the number of net days with work disability benefits per 30-day period. In order to not introduce bias regarding unemployed people (since information on their benefits are available already from day 2), only new sick-leave spells lasting more than 14 days were included in calculations for the following months. Six people died after 180 days but before end of follow-up. They were treated as having 0 days of work disability per month after death.

In the next type of analyses, once the optimal number of groups was computed, the individual probabilities of belonging to a particular group were estimated using a multinomial logit function. Individuals were assigned to the group that they had the greatest probability of belonging to. χ^2^ tests were used to compare the groups regarding sociodemographics and morbidity. We calculated the χ^2^ value separately for each diagnosis group regarding sick-leave diagnoses and specialised outpatient and inpatient diagnoses. To find how much each covariate explains variance in trajectory groups, we used multinomial logistic regression and calculated the difference between the Nagelkerke r^2^ of the full model and a model without the covariate of interest. Again, we did this for each covariate, running models for each diagnosis group separately. Since the Nagelkerke r^2^ is a pseudo-r^2^, caution is needed when interpreting these results.

The project was approved of by the Regional Ethical Review Board of Stockholm. All analyses were done in SAS v 9.3 (SAS Institute, Cary, NC).

## Results

The majority of the cohort were women, had more than elementary education, and were in paid work when the sick-leave spell begun. Nearly half were married or cohabitating and the median age was 42. The majority were born in Sweden and 10% outside the EU (Table 1).

The index sick-leave spell became at least 180 days long for 28.7% of the cohort. This proportion was lower among the younger (16–24 years) and the employed and higher among those born outside the EU and those unemployed.

**Fig. 1:**
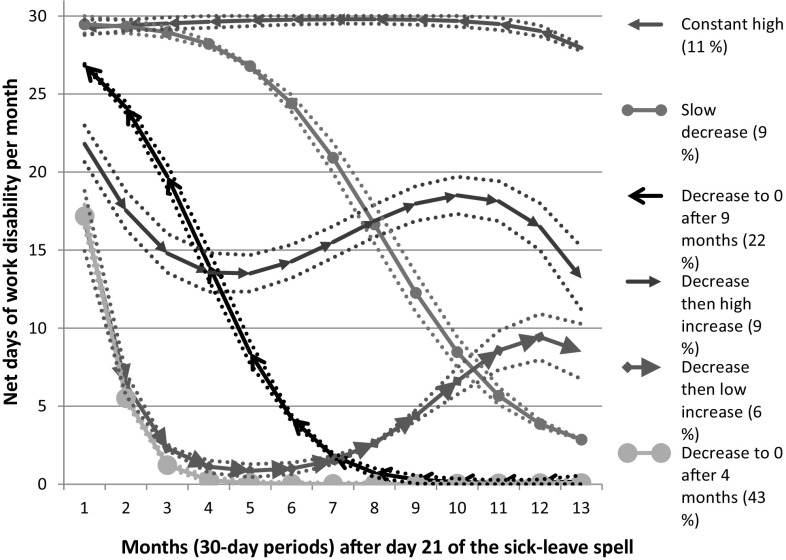
Group-based trajectories for mean number of net days/30 day period of work disability (sick leave and disability pension) per month (with 95% CI) during 13 months from day 21 of the index sick-leave spell

We identified six trajectories of future work disability and named them after the progression of work disability days per month. With seven groups, even though the BIC value was closer to 0, one group had less than 5% of the study population, and the confidence intervals (CI) became overlapping for two other groups, meaning that distinguishing between the groups and assigning group membership with at least 0.70 average probability became difficult. Therefore, the model with six groups was chosen as the most appropriate to describe the population. Each individual was assigned to the group she or he had the highest probability of belonging to. All groups had an average probability of above 0.90, indicating good model fit. Figure 1 shows the trajectories and the 95% CI for each curve, as well as the names given them.

The largest group (43% of all) had no or almost no work disability days/month after month four, and was, therefore, called “decrease to 0 after 4 months”. Another group (6%) also decreased to almost 0 by month four, but this group then increased their work disability again (“decrease then low increase”). The CIs for the group “decrease then low increase” were wide at the end of follow up, indicating a large spread of values within the group. The second largest group (22%) also decreased the average number of work disability days per month, although this group started at a higher average in month 1 and only reached 0 days in month 9 (“decrease to 0 after 9 months”). There were two groups that started their sick leave with an average of 30 days of work disability per month in the first 2 months, but for one of these groups, the average number of work disability days/month decreased (“slow decrease”, 9%), whereas it remained high for the other (“constant high”, 11%). The final group had decreasing work disability in the first 3 months of the study, then increasing, and finally decreasing again in the final months (“decrease then high increase”, 9%). The groups who ended the study period with no work disability days (“decrease to 0 after 4 months” and “decrease to 0 after 9 months”) together made up about 65% of the cohort.

The group “decrease to 0 after 4 months” (43%) was characterised by the highest proportion of people with no sick leave prior to the index spell and lowest proportion of specialist out- or inpatient care, along with the group “decrease then low increase”. The group “decrease to 0 after 4 months” also had the highest proportion of younger individuals (< 35 years), along with the group “decrease to 0 after 9 months” (Tables 2, 3).

**Table 2:** Proportion (%) of sociodemographic characteristics in each of the six different work disability trajectory groups and differences between the groups, in p-values, plus Nagelkerke r^2^ with variable removed from model and difference to Nagelkerke r^2^ of full model

	Constant high	Slow decrease	Decrease to 0 at 9 months	Decrease then high increase	Decrease, then low increase	Decrease to 0 at 4 months	χ^2^-test p-value	Nagelkerke r^2^	Difference in Nagelkerke r^2^
Sex									
Women	64.6	68.6	68.3	68.0	71.3	69.0	0.059	0.1583	0.0000
Men	35.4	31.4	31.7	32.0	28.7	31.0			
Age (years)									
16–24	5.7	5.0	6.5	3.3	5.4	6.7	< 0.001	0.1534	0.0049
25–34	21.3	23.8	26.1	19.8	20.0	23.9			
35–44	29.1	32.6	30.3	31.9	30.9	29.4			
45–54	28.2	24.6	24.0	27.2	24.7	24.9			
55–64	15.8	14.0	13.1	17.8	19.0	15.2			
Country of birth									
Sweden	78.0	87.2	85.6	83.8	83.1	85.2	< 0.001	0.1552	0.0032
Other Nordic country	3.1	2.0	2.6	2.9	3.9	3.2			
EU25 without Nordic countries	3.3	1.8	1.9	2.6	2.7	2.7			
Rest of the world	15.6	9.0	9.8	10.7	10.3	8.9			
Education									
Elementary (≤ 9 years)	18.6	13.3	11.6	11.7	13.9	12.9	< 0.001	0.1555	0.0029
Secondary (10–12 years)	52.4	48.5	51.3	46.4	54.1	49.3			
Post-secondary (> 12 years)	29.0	38.1	37.1	42.0	32.0	37.8			
Family situation									
Married/living with partner without children	8.9	9.9	10.5	11.6	10.6	10.5	0.168	0.1571	0.0013
Married/living with partner with children	34.3	39.4	38.0	35.4	35.0	38.0			
Single/divorced/separated/widowed without children	40.2	33.9	36.5	36.4	38.0	36.7			
Single/divorced/separated/widowed with children	16.4	16.5	14.7	16.4	16.0	14.2			
Living with parents (≤ 20 years old)	0.3	0.3	0.3	0.2	0.4	0.5			
Marital status									
Single, divorced, widowed	65.4	62.3	60.5	59.1	63.7	61.3	0.038	0.1575	0.0008
Married, registered partnership	34.6	37.7	39.5	40.9	36.3	38.7			
Type of living area									
Big cities (Stockholm, Gothenburg, Malmö)	42.7	40.3	41.0	39.7	39.2	38.4	0.033	0.1567	0.0017
Medium-sized town (> 90,000 inhabitants)	32.1	33.2	33.4	35.8	32.1	36.6			
Small towns/village (< 90,000 inhabitants)	25.2	26.6	25.6	24.5	28.7	25.0			
Employment status									
Employed	60.6	84.1	84.4	87.2	89.1	88.2	< 0.001	0.1276	0.0307
Unemployed	33.5	10.3	9.5	6.8	6.4	6.9			
Self-employed/student	3.7	4.0	3.4	4.2	3.6	2.7			
Parental leave	2.2	1.6	2.6	1.8	0.9	2.2			

**Table 3:** Proportion (%) of sick leave and healthcare consumption characteristics in each of the six trajectory groups among people sickness absent due to depressive episode (F32) ≥ 21 days and respective p-values, plus Nagelkerke r^2^ with variable removed from model and difference to Nagelkerke r^2^ of full model

	Constant high	Slow decrease	Decrease to 0 at 9 months	Decrease then high increase	Decrease, then low increase	Decrease to 0 at 4 months	χ^2^-test p-value	Nagelkerke r^2^	Difference in Nagelkerke r^2^
Full- or part-time sick leave at start of index sick-leave spell									
Part-time 25–75%	3.5	3.5	11.2	27.2	19.3	19.6	< 0.001	0.122	0.0364
Full-time 100%	96.5	96.5	88.8	72.8	80.7	80.4			
Sick-leave days in preceding 365 days									
No days	64.2	73.3	72.3	60.3	68.0	76.6	< 0.001	0.1498	0.0085
0.25–49.75 days	17.0	15.3	18.1	21.9	20.9	16.3			
50–89.75 days	5.1	3.9	4.1	7.0	4.9	3.9			
90–179.75 days	6.8	4.2	3.8	7.5	4.9	2.4			
180–365 days	6.9	3.3	1.7	3.3	1.2	0.8			
Previous sick-leave diagnosis									
Depressive episode (F32)	11.5	7.9	9.5	13.8	11.8	8.1	< 0.001	0.1575	0.0008
Other mental diagnoses^a^	9.4	7.5	7.7	11.5	8.2	6.0	< 0.001	0.1579	0.0004
Somatic diagnoses^b^	21.8	14.2	15.3	20.3	15.7	13.0	< 0.001	0.1574	0.001
Purchases of prescribed antidepressants previous to sick leave									
No purchases	57.7	53.1	54.4	57.3	53.8	56.9	0.011	0.1572	0.0011
1–2 purchases	41.2	46.3	45.1	41.8	45.7	42.7			
3 or more puchases	1.1	0.6	0.4	0.9	0.4	0.4			
Purchases of prescription antidepressants during first 21 days									
No purhcases	47.0	60.1	59.6	48.2	52.2	62.1	< 0.001	0.1523	0.0061
1–2 purchases	21.5	19.9	20.4	20.6	18.7	19.4			
3 or more purchases	31.5	20.0	20.0	31.2	29.1	18.6			
Previous outpatient specialist visits									
No visits	46.4	48.7	52.6	48.6	52.9	55.9	< 0.001	0.1573	0.0011
1 visit	17.1	21.2	19.6	19.8	19.1	20.2			
2–3 visits	19.2	18.2	15.6	15.9	16.7	14.3			
4 or more visits	17.3	11.9	12.2	15.6	11.2	9.6			
Specialized outpatient visit diagnosis									
Depressive episode (F32)	10.9	5.8	5.1	6.1	6.3	4.1	< 0.001	0.1582	0.0002
Other mental diagnosis^a^	14.0	7.9	9.0	10.8	8.5	6.2	< 0.001	0.1581	0.0003
Somatic diagnosis^b^	44.3	45.7	42.1	46.1	40.5	40.0	< 0.001	0.1581	0.0003
Outpatient specialist visit in the first 21 days									
No visit	76.1	80.5	81.3	82.2	83.1	85.6	< 0.001	0.1579	0.0004
1 visit	17.3	13.8	13.1	12.5	10.6	10.5			
2 or more visits	6.7	5.7	5.6	5.3	6.3	3.8			
Specilized outpatient visit diagnoses									
Depressive episode (F32)	10.4	5.8	6.3	5.7	6.1	4.8	< 0.001	0.1579	0.0004
Other mental diagnosis^a^	5.7	5.2	4.6	5.0	4.2	3.3	0.002	0.1581	0.0003
Somatic diagnosis^b^	9.8	10.2	9.9	9.1	8.1	7.9	0.031	0.1575	0.0008
Previous inpatient care									
No inpatient care	83.2	88.8	89.5	85.0	90.7	90.2	< 0.001	0.1582	0.0002
At least one day	16.8	11.2	10.5	15.0	9.3	9.8			
Diagnosis for inpatient care									
Depressive episode (F32)	2.9	1.0	1.3	1.6	0.7	1.0	< 0.001	0.1581	0.0002
Other mental diagnosis^a^	4.3	2.0	2.4	4.8	1.6	1.6	< 0.001	0.1575	0.0009
Somatic diagnosis^b^	12.0	8.9	7.7	11.0	7.5	7.8	< 0.001	0.1581	0.0002
Suicide attempt^c^	1.7	0.8	0.5	0.8	1.0	0.7	0.022	0.1577	0.0007
Inpatient care during first 21 days									
No inpatient care	88.6	92.8	91.7	91.5	94.6	94.5	< 0.001	0.1575	0.0008
At least one day	11.4	7.2	8.3	8.5	5.4	5.5			
Diagnosis									
Depressive episode (F32)	5.3	3.2	4.6	3.9	2.1	2.3	< 0.001	0.1573	0.0011
Other mental diagnosis^a^	4.8	2.5	2.5	2.8	1.5	2.1	< 0.001	0.1575	0.0008
Somatic diagnosis^b^	2.7	2.5	2.4	2.7	1.9	1.8	0.282	0.1576	0.0008
Suicide attempt^c^	1.2	1.0	0.9	0.7	0.9	0.6	0.359	0.1579	0.0005

^a^F00-99, excluding F32

^b^All diagnoses excluding F00-99, O80, Z00-99

^c^X60–X84

The group “decrease then low increase” (6%) was characterised by the lowest proportion of specialized out- or inpatient care, along with the group “decrease to 0 after 4 months” (Table 3).

The “slow decrease” group had the highest proportion of individuals born in Sweden. This group also had the highest proportion of those individuals who were on full-time sick leave at the start of their spell, along with the “constant high group” (97% in both groups), and thus had the highest total number of work disability days per month during the first months. The group “slow decrease” was also characterised by a high proportion of outpatient care due to somatic diagnoses (Tables 2, 3).

The “constant high” (11%) group was distinct by its higher proportion of men, individuals born outside of the Nordic countries, individuals with only elementary education, and a substantially higher proportion of unemployed than the other groups. There was also a higher proportion of healthcare consumption, both before the index sick-leave spell and during the first 21 days of the index spell in this group. This was especially prominent regarding care due to depression or another psychiatric diagnosis. This group also had a higher proportion of people who had received inpatient care due to suicide attempts, both prior to and during the first 21 days of the sick-leave spell (Tables 2, 3).

The “constant high” group was also characterised by the highest proportion of people with prior sick leave, along with the group “decrease, then high increase”. These two groups (“constant high” and “decrease then increase”) were the groups with the highest proportion of previous depression-related sick leave, yet they had the highest proportion of people who had not purchased any prescription antidepressants prior to the index sick-leave spell. However, these two groups had a somewhat higher proportion of having purchased prescription antidepressants three or more times during the first 21 days of the index sick-leave spell, than other groups. In all groups, only a very small proportion of individuals had three or more purchases of prescribed anti-depressants in the year prior to the index sick-leave spell (Table 3).

The group “decrease then high increase” (9%) was characterised by the highest proportion of part-time sick leave at the start of the spell, and the highest proportion of prior sick leave due to mental diagnoses; depression and others. This group was also characterised by a high proportion of specialist in- and outpatient care due to somatic diagnosis (Table 3).

In all groups, a higher proportion had had previous sick leave with a somatic diagnosis than to depressive episode or another mental diagnosis.

The Nagelkerke r^2^ for the full model was 0.1583 (not shown in table). The variables where the difference between the full model r^2^ and the model without the covariate of interest was greatest were extent of sick-leave at start of index spell (full or part-time), with a difference of 0.0364, and employment status at the start of the index spell, with a difference of 0.0307. These were, thus, the two variables with the most explanatory power over group membership in our analysis.

## Discussion

In this exploratory prospective cohort study of future sick-leave trajectories among all individuals with a new sick-leave spell ≥ 21 days due to depressive episode, we identified six different work disability trajectories: “constant high”, “decrease then high increase”, “slow decrease”, “decrease then low decrease”, “decrease to 0 after 9 months” and “decrease to 0 after 4 months”. 65% had no work disability (sick leave or disability pension) at the end of follow up, 13 months later. There were some distinct differences between the people of the different trajectories regarding sociodemographic and morbidity characteristics. For example, those groups who reduced their sick leave to 0 days/month by the end of the study period had a higher proportion of younger individuals while the groups “constant high” and “decrease then high increase” had the highest proportion of people with prior sick leave in the year before the index spell. Factors such as previous sick leave, comorbidity (mental and somatic), purchase of prescription antidepressants, employment status, birth country, and educational level, would need to be included when aiming to predict duration of a sick-leave spell.

The recommendation from the Swedish National Board of Health and Welfare is that in many cases a depressive episode does not need to lead to sick leave at all [54]. If sick leave is needed, it should not be longer than 4 months for mild severity, and not longer than 6 months for severe cases. Hence it is notable and concerning that only 43% of the study population were not on sick leave or disability pension at 4-months follow-up and that 35% of the cohort were still on either sick leave or disability pension after 13 months.

We showed that it is a process to come off from work disability, with progressively fewer work disability days each month for those who end the study with no work disability. We also showed that the pace of this varies. Some people reduce their work disability in ways similar to those who reduce it to 0, only to increase it again: by picking an end of follow-up and seeing whether the individuals were still on work disability or studying time to return to work, we would have missed this pattern. A systematic review found inconsistent results for most of the studied risk factors of long sick-leave spells, and that there was only consistent evidence to identify old age and a previous history of morbidity and of sick leave as risk factors [13]. These inconsistencies could be due to confounding from different definitions of a long sick-leave spell [55].

We were also interested in how individuals in the different trajectory groups differed regarding sociodemographic and morbidity characteristics.

The “constant high” group was distinct from other groups on a number of sociodemographic characteristics (e.g., age, country of birth, education, employment status) identified as risk factors of morbidity [56] and of sick leave [57]. Individuals in this group also tended to have more indicators of a more severe case of morbidity regarding previous sick leave, antidepressant consumption, and inpatient and specialized outpatient healthcare both prior to and during the 21 first days of the sick-leave spell. Severity of symptoms has been found to be associated with sick-leave duration in several studies [35, 58, 59], while one small study did not find a significant association [60].

Regarding indicators of morbidity, specifically sick leave and healthcare, in the “decrease, then high increase” group, a greater proportion had part-time sick leave at the start of the index spell, and more previous sick leave with mental and somatic diagnoses other than depressive episode. This could indicate a pattern of recurring sick leave in this group both before and during the study period, that warrants further investigations. Issues to investigate are e.g., whether they returned to work too early or if their subsequent sick leave was due to other somatic or mental diagnoses. This group tended to have somewhat higher rates of healthcare use due to a somatic diagnosis, indicating that their depression was comorbid with another condition. Previous research has found that return to work is often delayed if depression is comorbid with other somatic and mental conditions [34], and that return to work and symptom recovery are only moderately related: individuals can both return to work before they are fully recovered, and recover without returning to work [61]. Patients often find it difficult to know when the time is right to return to work, and the decision can itself prompt further stress and anxiety [62].

We identified six different trajectories and some characteristics that distinguish the groups from each other. However, this is a first, exploratory study, and the groups would need to be studied more in depth, for example, regarding comorbidities, or regarding working conditions and work content.

### Methodological Considerations

A main strength of this study is that it was based on the entire population, not a sample, covering all people of working age in Sweden. Other strengths were the large cohort, allowing for subgroup analysis, that information on a wide variety of sociodemographic and morbidity aspects, could be included from several high-quality nationwide registers [63, 64], that all information was register based, that is, not self-reported, and that there was no loss to follow up.

We excluded those who had reached the maximum number of sick-leave days during the year prior to the index spell and those who were on part-time disability pension when the sick-leave spell started, as both of these groups already have a well-known risk of long-term sick leave and as part-time disability pension is not a possibility in most countries.

By including all sick-leave spells ≥ 21 days, we eliminated any bias that stems from the differing rules applied to when individuals can be granted sick-leave benefits from the Social Insurance Agency. However, this means that those with sick-leave spells with a shorter duration were not included in the study.

We combined gross days into net days so that 2 days of 50% would be the same as 1 day of 100% absence, which might be problematic for the interpretation of results. However, since people can have part-time disability pension while also having part-time sick leave, not combining them would mean that some people would have more than 30 days absence per month, which would also have been problematic for the interpretation of the results. Using gross days would also mean that a day with 25% absence would be equalled to one with 100% absence.

It is possible that the diagnosis depressive episode (ICD-10 F32) was used sometimes when the diagnosis recurring depression (ICD-10 F33) should have been more appropriate. About 10% of the cohort had a sick-leave spell due to depressive episode in the 365 days before the index spell, despite that the guidelines from the National Board of Health and Welfare state that the diagnosis recurring depression (F33) should be used for recurring depressive periods. The guidelines from the National Board of Health and Welfare are roughly similar concerning depressive episode and recurring depression (up to 6 months for severe cases), although severity of depression can often increase with each recurrence [54]. This means that it is possible that some individuals with longer sick-leave spells should in fact have had the diagnosis recurring depression.

A topic of frequent discussion in this research field is the validity of sick-leave diagnoses, however, this is seldom studied. A study conducted in Sweden in 1991 showed high validity of sick-leave diagnoses compared with diagnoses from medical records [65]. Only the main sick-leave diagnosis is registered in the Social Insurance Agency’s MiDAS database, that is, the diagnosis that the sickness certifying physician judges to contribute most to the reduced work capacity. When depressive episode is comorbid with other diseases, it might be difficult to determine when depression is the main condition and when it is secondary to other somatic or mental disorders in affecting work capacity [66]. This means that we have not included sick-leave spells where depressive episode has been included as a contributing factor only, but not the main cause. Furthermore, in the MiDAS register the Social Insurance Agency only registers the diagnosis at the ICD10 three-digit level. It would, of course have been an advantage to also have the fourth digit, as there are differences between the diagnoses included in this group, especially regarding depression severity. Such differences between the diagnoses in the F32 diagnosis group may explain some of the differences in future SA trajectories that we observed. We used information about specialist out- and inpatient healthcare as a proxy indication of disease severity, given the lack of this information in the diagnosis. However, it is not certain that a more severe diagnosis leads to longer SA duration. The SA duration is instead linked to how soon the patient gets optimal treatment, how soon the depressive symptoms decrease and on their impact on function and work capacity.

The registers used in this study only contain information on sociodemographic, sick leave, specialised outpatient and inpatient care, and purchase of prescribed medication. There are many other factors that also might be associated with length of sickness absence, such as work-related or personality factors, however, for information on those, additional types of data is needed.

## Conclusion

After an initial sick-leave spell lasting at least 21 days with a diagnosis of depressive episode, we identified six different trajectories of future work disability that individuals could belong to. The majority of the study population had no work disability by the end of the study period. There were differences regarding sociodemographic and morbidity characteristics between the identified groups, which could be used to identify the most likely trajectory of a sick-leave spell.
